# Genome-Wide Identification of Regulatory Elements and Reconstruction of Gene Regulatory Networks of the Green Alga *Chlamydomonas reinhardtii* under Carbon Deprivation

**DOI:** 10.1371/journal.pone.0079909

**Published:** 2013-11-01

**Authors:** Flavia Vischi Winck, Samuel Arvidsson, Diego Mauricio Riaño-Pachón, Sabrina Hempel, Aneta Koseska, Zoran Nikoloski, David Alejandro Urbina Gomez, Jens Rupprecht, Bernd Mueller-Roeber

**Affiliations:** 1 GoFORSYS Research Unit for Systems Biology, Institute of Biochemistry and Biology, University of Potsdam, Potsdam-Golm, Germany; 2 GoFORSYS Research Unit for Systems Biology, Max-Planck Institute of Molecular Plant Physiology, Potsdam-Golm, Germany; 3 Group of Computational and Evolutionary Biology, Biological Sciences Department, Universidad de los Andes, Bogotá, Colombia; 4 University of Potsdam, Institute of Physics, Potsdam-Golm, Germany; 5 Systems Biology and Mathematical Modeling Group, Max-Planck Institute of Molecular Plant Physiology, Potsdam-Golm, Germany; 6 Potsdam Institute for Climate Impact Research (PIK), Potsdam, Germany; 7 Department of Physics, Humboldt University of Berlin, Berlin, Germany; Universidad Miguel Hernández de Elche, Spain

## Abstract

The unicellular green alga *Chlamydomonas reinhardtii* is a long-established model organism for studies on photosynthesis and carbon metabolism-related physiology. Under conditions of air-level carbon dioxide concentration [CO_2_], a carbon concentrating mechanism (CCM) is induced to facilitate cellular carbon uptake. CCM increases the availability of carbon dioxide at the site of cellular carbon fixation. To improve our understanding of the transcriptional control of the CCM, we employed FAIRE-seq (formaldehyde-assisted Isolation of Regulatory Elements, followed by deep sequencing) to determine nucleosome-depleted chromatin regions of algal cells subjected to carbon deprivation. Our FAIRE data recapitulated the positions of known regulatory elements in the promoter of the *periplasmic carbonic anhydrase* (*Cah1*) gene, which is upregulated during CCM induction, and revealed new candidate regulatory elements at a genome-wide scale. In addition, time series expression patterns of 130 transcription factor (TF) and transcription regulator (TR) genes were obtained for cells cultured under photoautotrophic condition and subjected to a shift from high to low [CO_2_]. Groups of co-expressed genes were identified and a putative directed gene-regulatory network underlying the CCM was reconstructed from the gene expression data using the recently developed IOTA (inner composition alignment) method. Among the candidate regulatory genes, two members of the MYB-related TF family, *Lcr1* (*Low-CO*_*2*_ *response regulator 1*) and *Lcr2* (*Low-CO*_*2*_ *response regulator 2*), may play an important role in down-regulating the expression of a particular set of TF and TR genes in response to low [CO_2_]. The results obtained provide new insights into the transcriptional control of the CCM and revealed more than 60 new candidate regulatory genes. Deep sequencing of nucleosome-depleted genomic regions indicated the presence of new, previously unknown regulatory elements in the *C. reinhardtii* genome. Our work can serve as a basis for future functional studies of transcriptional regulator genes and genomic regulatory elements in *Chlamydomonas*.

## Introduction

Like other green algae, *Chlamydomonas reinhardtii* has evolved an inorganic carbon (Ci) concentrating mechanism (CCM) to support photosynthetic carbon fixation. Through this mechanism, the availability of CO_2_ is increased in the vicinity of the carbon fixation enzyme, ribulose-1, 5 bisphosphate carboxylase oxygenase (Rubisco), favoring photosynthesis [1,2]. The CCM is important for survival in niches where the concentration and diffusion of CO_2_ is low or Ci depletion can occur rapidly, e.g., at the surface of aquatic environments [3–7]. In particular, CCM is induced when the extracellular concentration of Ci declines to below 0.5% [8]. Several genes, including *low-CO*_*2*_ *-inducible chloroplast envelope* protein *1* (*Ccp1*), *low-CO*_*2*_ *-inducible chloroplast envelope* protein *2* (*Ccp2* ), *low-CO*_*2*_ *inducible* protein *1* (*Lci1*), *High-light activated 3 protein* (*Hla3*) and *Rhesus* protein *1* (*Rh1*), have been suggested to encode membrane proteins that function as HCO_3_^-^ transporters and CO_2_ channels in the plasma membrane and the chloroplast outer envelope membrane, thereby participating in the CCM [9,10]. Studies on the cyanobacterium *Synechocystis* PCC6803 and the green alga *Chlamydomonas acidophila* have shown that cells cultivated at low-CO_2_ (LC) concentration in the light take up Ci more rapidly than cells cultivated at LC in the dark, suggesting a cooperative effect involving light and carbon signaling in the modulation of the expression of CCM-related genes [11,12]. Similar findings were also reported for *C. reinhardtii* where light in addition to a decrease in Ci concentration is needed to activate the CCM [13]. Previous studies with *Chlamydomonas* mutants led to the identification of regulatory genes, including *Lcr1* (*low-CO*_*2*_ *response regulator 1*), which encodes a transcription factor (TF) of the MYB-related family [14,15] and was suggested to recognize genomic regulatory regions and control the transcription of genes involved in CCM. In *Lcr1* mutants, the expression of CCM-related genes, including *periplasmic carbonic anhydrase1* (*Cah1*) [13] and *Lci1*, is reduced [14]. It was suggested that Lcr1 translocates to the nucleus where it binds the *Cah1* promoter, thereby regulating *Cah1* transcription in response to low Ci concentration [15]. The enzyme carbonic anhydrase equilibrates the cellular concentrations of CO_2_ and HCO_3_^-^ and represents an important component of the CCM [1]; however, there is currently no complete understanding of the gene regulatory network underlying the CCM. 

A Ci uptake-deficient mutant was previously isolated in *Chlamydomonas* and the gene underlying the mutation - *Ccm1* - was cloned. *Ccm1* encodes a hydrophilic protein that harbors a C2H2-type zinc-finger motif likely conferring DNA binding property. Interestingly, the cellular level of Ccm1 does not change during the acclimation to LC condition, indicating post-translational control of Ccm1 activity [8]. 

In order to gain a deeper understanding of the regulatory mechanism underlying the CCM in *C. reinhardtii* and to identify possible regulatory regions and new regulators of this physiological process we carried out Formaldehyde-assisted Isolation of Regulatory Elements (FAIRE) which allowed us to disclose potential regulatory elements of CCM-related genes. In addition, we observed the expression of 130 genes encoding transcription factors and other transcriptional regulators (TRs), which we had previously predicted [16,17] on the basis of the *C. reinhardtii* genome sequence [18]. Expression patterns were determined for cells grown photoautotrophically in constant light and subjected to a shift from high CO_2_ (HC) to LC concentration. Next, in order to infer the regulatory interactions between genes from the time-resolved data, we employed a permutation-based method (called IOTA), which is computationally efficient, robust against noise, applicable to short time series data, and capable of reconstructing the directionality of the regulatory interactions [19]. Thus, based on the obtained gene expression data and its analysis, novel candidate regulatory regions and genes involved in controlling CCM were uncovered and a gene regulatory network (GRN) underlying CCM was reconstructed. 

## Results

### Induction of CCM verified by marker genes

Previous reports demonstrated enhanced expression of *Cah1* and mitochondrial carbonic anhydrase (Mca) during CCM induction in *Chlamydomonas* wild-type strains cultured in photoautotrophic conditions [7,14]. Similarly, our study revealed enhanced expression of both genes in *Chlamydomonas* cells cultured at LC concentration (Figure **S1**), indicating that CCM was indeed induced in our experimental setting. However, *Mca* expression was not as strongly up-regulated as it was in other previous experiments. The differences in induction levels may be due to the fact that in the previous experiments different *Chlamydomonas* strains and experimental conditions were used.

### Putative regulatory elements of the Cah1 gene

**Figure 1:**
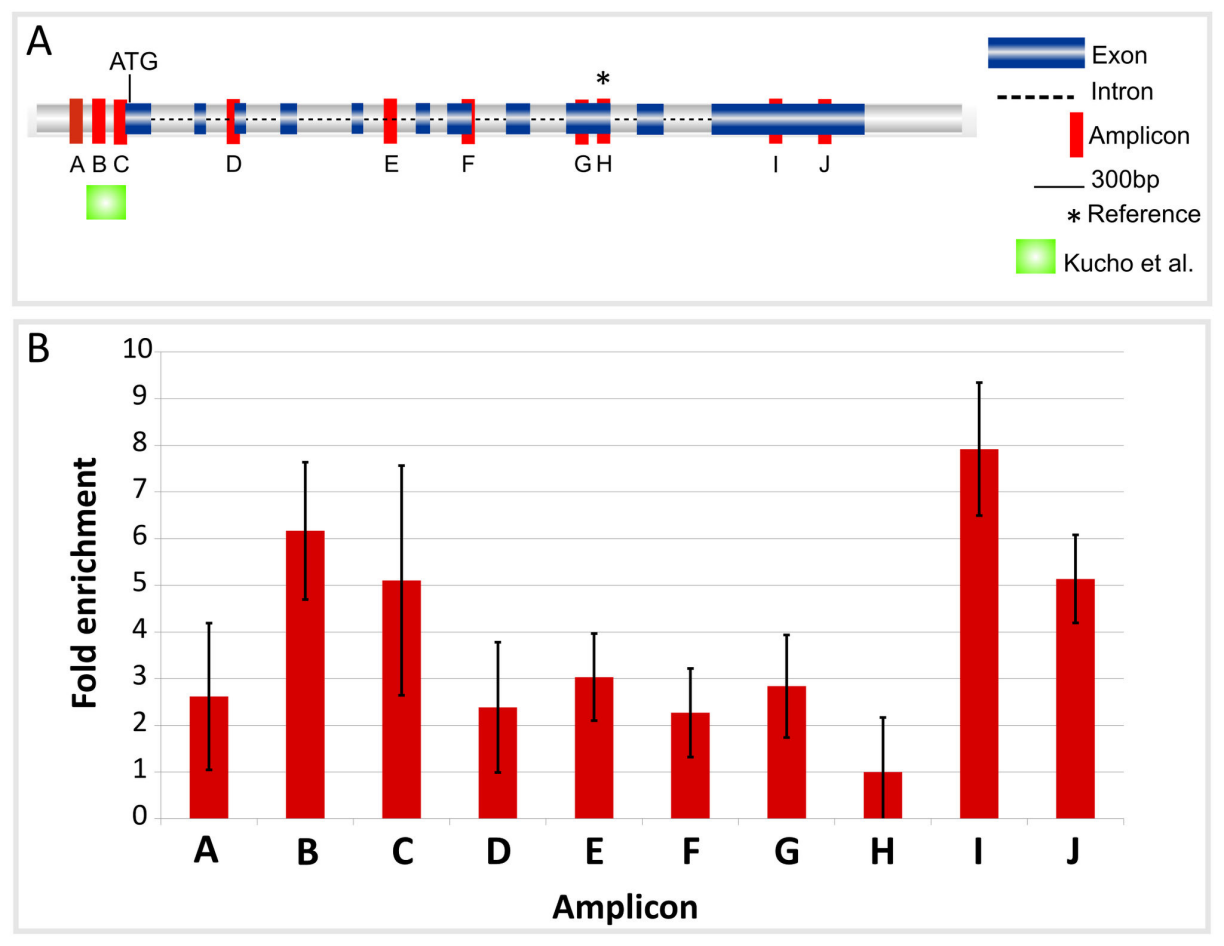
Quantitative PCR of FAIRE fragments derived from the *Cah1* locus. (A) Quantitative PCR analysis was performed for ten DNA segments of the *Cah1* locus and enrichment of each fragment was measured. Red areas show the location of the DNA segments amplified and quantified. Exons are indicated by blue boxes and introns by dashed lines. (B) DNA segments located in the promoter region (amplicons B and C) and in a region located in the last exon of the *Cah1* gene, representing the 3´ UTR (amplicons I and J), were found to be enriched. De-crosslinked chromatin was used as control for calculating the enrichment of genomic fragments in the FAIRE samples. The enrichment was determined as 2^-ΔΔCt^ (cf. Material and Methods) and error bars indicate the standard deviation (SD) from three biological replicates. The reference region H is indicated by a star (*) in (A) and refers to the region where the C_t_ value ratio between FAIRE and control samples was the lowest. The C_t_ values of the reference region were used for the calculation of the FAIRE enrichment as a normalization parameter. The position of regulatory elements of the LC response previously identified by Kucho et al. [24] is indicated.

**Figure 2:**
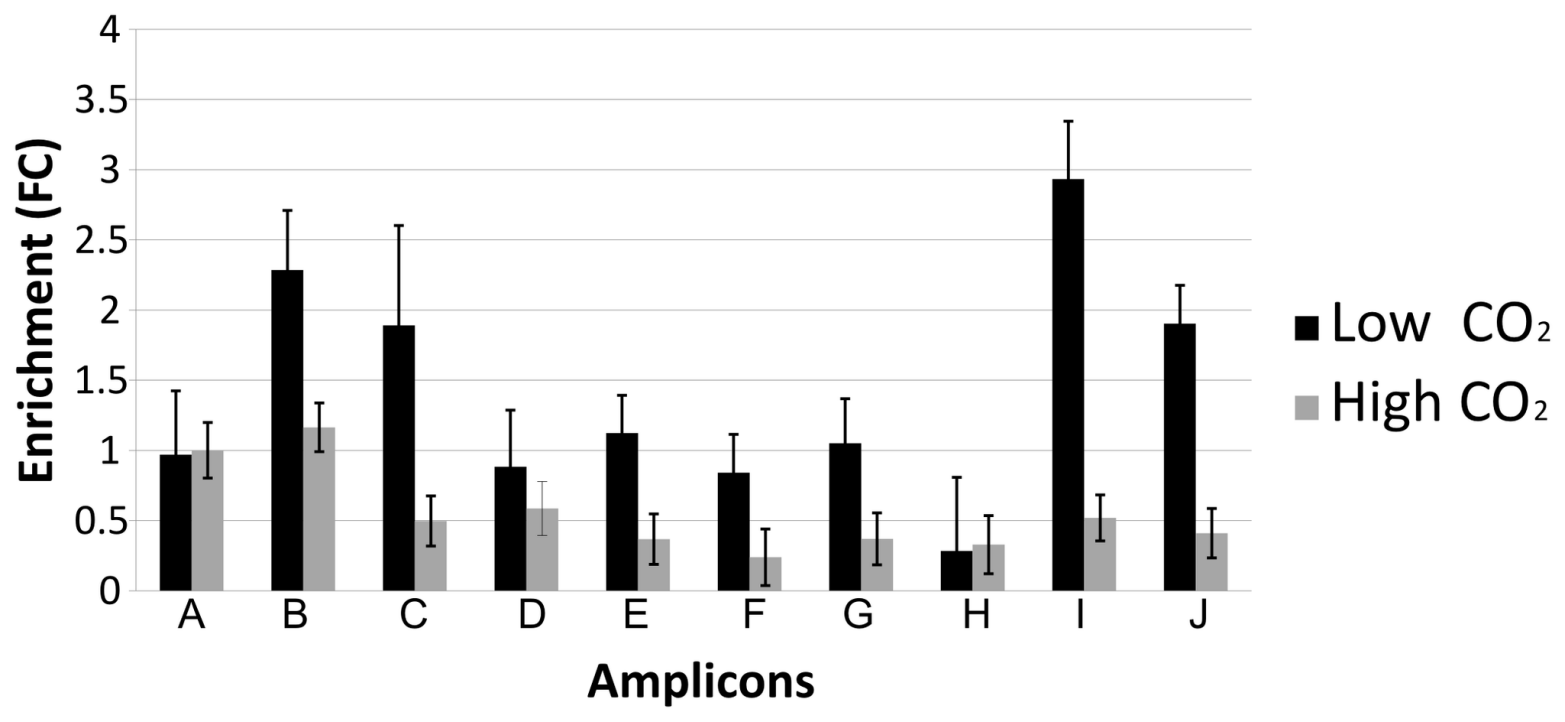
Comparison of enrichment of regulatory elements at the *Cah1* locus. Quantitative PCR analysis of FAIRE fragments was performed for the *Cah1* locus, with primers designed for covering coding and non-coding regions, represented by the amplicon identifier (Table 1). Enrichment of FAIRE fragments within the *Cah1* locus was analyzed by comparing the chromatin from cells cultured under HC and LC condition. Enrichment observed for the 5´ upstream and 3´ downstream regions of the gene locus is more pronounced in cells cultivated at LC, indicating that these regions may have regulatory functions.

In order to identify possible regulatory elements and their genome-wide distribution in *C. reinhardtii* we analyzed the nucleosome depletion pattern of the *Cah1* locus using the FAIRE method (cf. Materials and Methods). FAIRE samples were obtained from *C. reinhardtii* cells cultured at HC and for three hours in LC condition, and analyzed by quantitative PCR (qPCR) to check for enrichment of DNA fragments from different regions of the *Cah1* locus and its promoter (Figure **1A**). Primer pairs were designed to cover the coding and non-coding genomic regions of *Cah1*; qPCR revealed enrichment for DNA fragments located within the 5´-upstream region of *Cah1* (Figure **1B**, amplicons B and C) and in its downstream region (Figure **1B**, amplicons I and J). Notably, some of the enriched fragments were located in *Cah1* promoter segments containing previously identified regulatory elements of the LC response [20]. According to our results obtained with qPCR, a region depleted of nucleosomes is located within the first 300 bp upstream of the first ATG (translation start codon) of the *Cah1* transcript in cells cultured under LC condition. We compared the FAIRE-extracted DNA fragments of the *Cah1* locus isolated from cells harvested at HC and LC and found varied distribution of the enriched elements (Figure **2**), indicating dynamic behavior of the occupancy of regulatory elements involved in establishing the CCM. In addition, in cells cultivated at LC we observed an enriched DNA fragment in the downstream region of the *Cah1* gene locus (Figure **2**, amplicons I and J). The regulatory function of this region is so far unknown; however, our results confirm that the FAIRE method allows the identification of dynamic genomic regions likely to harbor regulatory sequences. Therefore, the enriched fragment located within the 3´-UTR of *Cah1* may function as an interaction site for DNA-binding proteins. 

### Genome-wide mapping of nucleosome-depleted chromatin segments revealed new candidate regulatory elements

**Figure 3:**
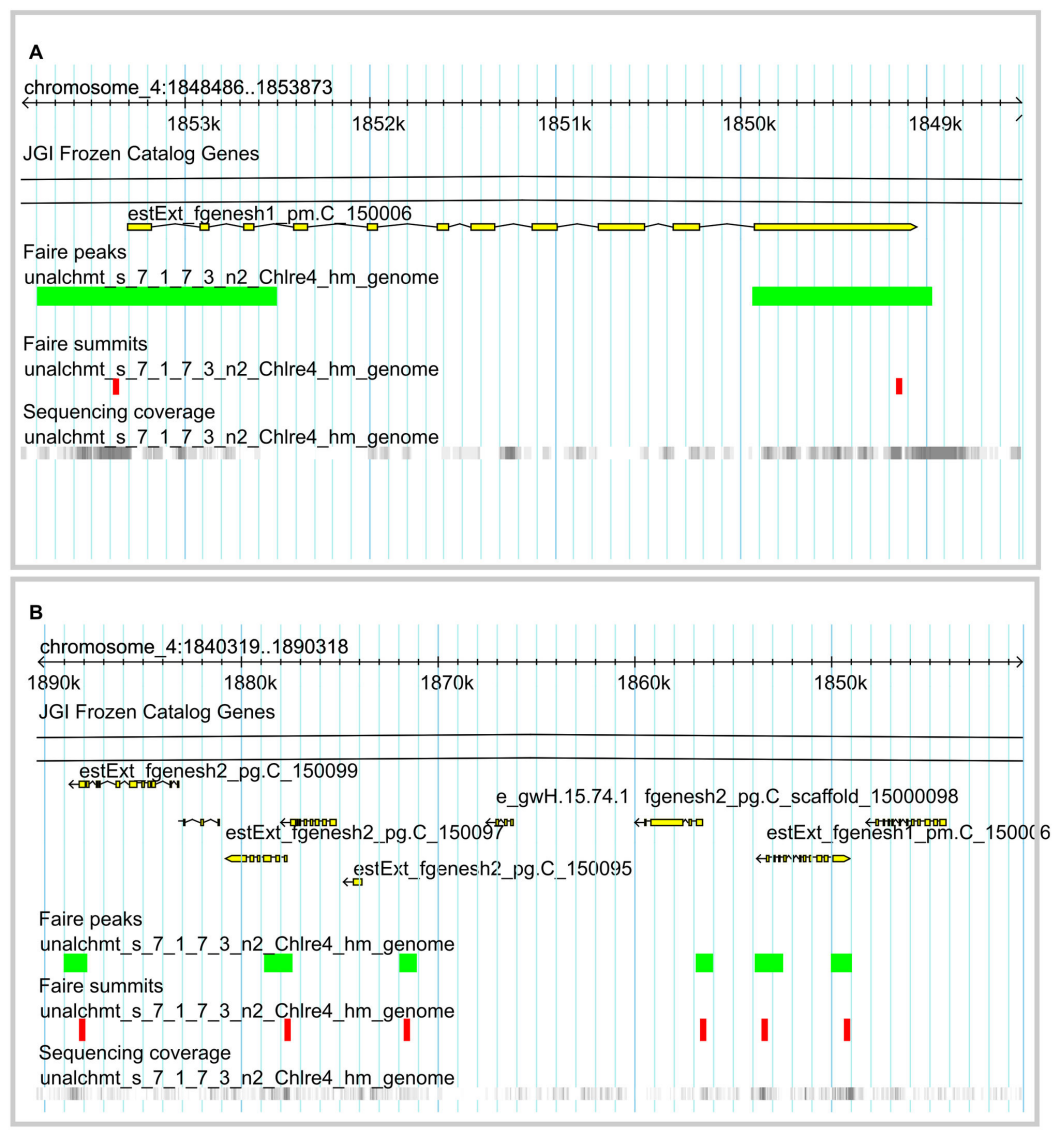
Web FAIRE-seq GenomeBrowser. FAIRE-seq reads mapped to the *Chlamydomonas* genome annotation v.4 were made accessible through a web GenomeBrowser (http://bce.uniandes.edu.co/gb2/gbrowse/Chlamydomonas_v4/). (A) *Cah1* locus. The annotated *Cah1* transcript is included; the yellow horizontal boxes indicate annotated exons and the connecting black lines indicate introns. Positions of FAIRE peaks are indicated as green horizontal bars, FAIRE summits as vertical red bars. Sequence coverage along the *Cah1* locus is indicated by the gray vertical bars; the darkness of the gray bars is proportional to the abundance of the sequencing reads mapped to this particular location. (B) Broader view of the genomic location where *Cah1* is annotated. Other transcripts in the proximity of the *Cah1* locus are shown, together with their corresponding features.

**Figure 4:**
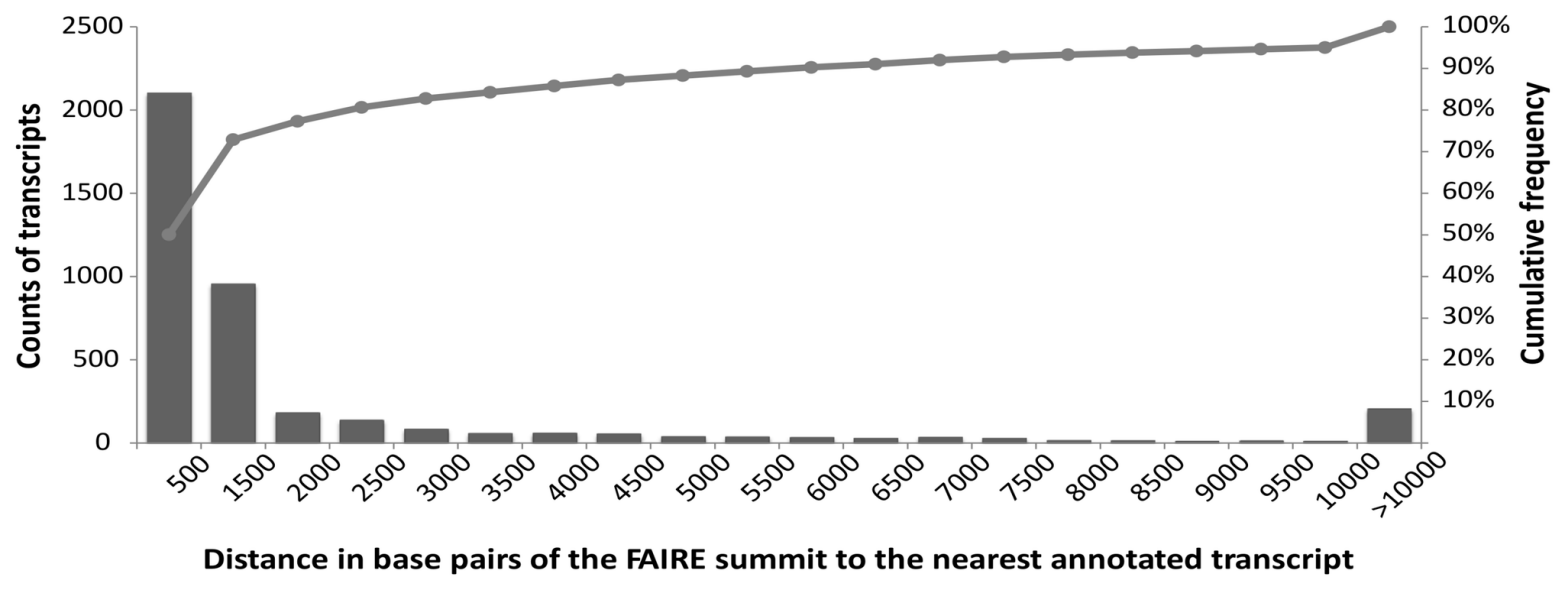
Cumulative histogram of the approximate distances of FAIRE summits from their next closest annotated gene. Genomic coordinates were used to calculate the distances between FAIRE summits and the start and stop codons of their nearest gene transcripts. The number of transcripts with at least one FAIRE peak assigned was counted and the distances to the start or stop codons computed. The gray line indicates the cumulative percentage of the transcripts to which FAIRE peaks were assigned. As seen, approximately 80% of the FAIRE summits were located within 1.5 kb up- or downstream of the transcript´s start or stop codon. FAIRE peaks were detected using MACS tool v. 1.4.0beta, with a *p*-value cutoff equal to 10^-05^.

The results from the qPCR analysis of the FAIRE samples prompted us to perform deep sequencing (FAIRE-seq) through which the entire set of putative regulatory elements involved in CCM induction may be identified. The DNA fragments obtained by FAIRE were sequenced using short-read paired-end technology (Illumina). The raw sequence data were deposited in the Sequence Read Archive (SRA) of the European Nucleotide Archive (ENA) (Acc: ERP001835; http://www.ebi.ac.uk/ena/data/view/ERP001835). The resulting sequence reads were mapped to the masked *Chlamydomonas* genome sequence v.4.0. Computational analysis performed with the MACS tool [21] revealed regions with significant (*p*-value < 10^-5^) tag enrichment (FAIRE peaks) and regions where the accumulation of these tags was maximal (FAIRE summits) along the genome (Figure **3**). In total, we discovered 6,442 FAIRE peaks along the *Chlamydomonas* genome, presented by a web genome browser (http://bce.uniandes.edu.co/gb2/gbrowse/Chlamydomonas_v4/). The presentation includes information about FAIRE peaks, FAIRE summits, sequence coverage, and annotated transcripts (http://genome.jgi-psf.org/Chlre4/download/FrozenGeneCatalog_20080828_ transcripts.fasta.gz) to show FAIRE peaks relative to their neighboring genes. Our findings indicate that nucleosome-depleted regions of the *C. reinhardtii* genome are preferentially localized in intergenic regions, in most cases close to transcription start sites (TSS) or to transcription termination sites (TTS). For the 6,442 FAIRE peaks identified, 4,206 FAIRE summits could be assigned to regions adjacent to gene coding sequences. The remaining FAIRE peaks could not be clearly assigned to any transcript and may therefore function as long-distant regulatory regions. These peaks were located in intergenic regions or in some cases within exons. We determined the distances between FAIRE summits and the 5´ and 3´ ends of gene coding sequences and observed that approximately 65% of them were located within 500-bp intervals up- or downstream of the translational start or stop codons. However, some of the FAIRE peaks were located at larger distances, up to thousands of base-pairs away from the next annotated open reading frame. A histogram showing the distribution of the FAIRE summits and the corresponding distances to gene coding sequences is plotted in Figure **4**. The presence or absence of FAIRE peaks close to transcripts was further investigated; we wanted to know whether FAIRE peaks close to coding sequences were associated with differential gene expression (Table **S1**). To this end, gene expression data obtained for TF and TR genes were used for the association analysis (cf. Materials and Methods). Our analysis indicated a significant association of the two variables (presence of a FAIRE peak and a more than 2-fold expression change, up or down; *p*-value = 0.005, chi-square test). Thus, FAIRE peaks were preferentially located in the vicinity of genes whose expression was more than 2-fold affected than close to genes that were only weakly affected by the treatment (here: shift from HC to LC concentration). We did, however, not observe any significant correlation between the presence of a FAIRE peak and the direction of the expression change (i.e., up- or down-regulation; *p*-value = 0.7). The same conclusion was previously drawn for nucleosome occupancy studies of gene promoters in yeast [22]. 

### Time-series gene expression analysis revealed TF and TR genes responsive to carbon dioxide limitation

**Figure 5:**
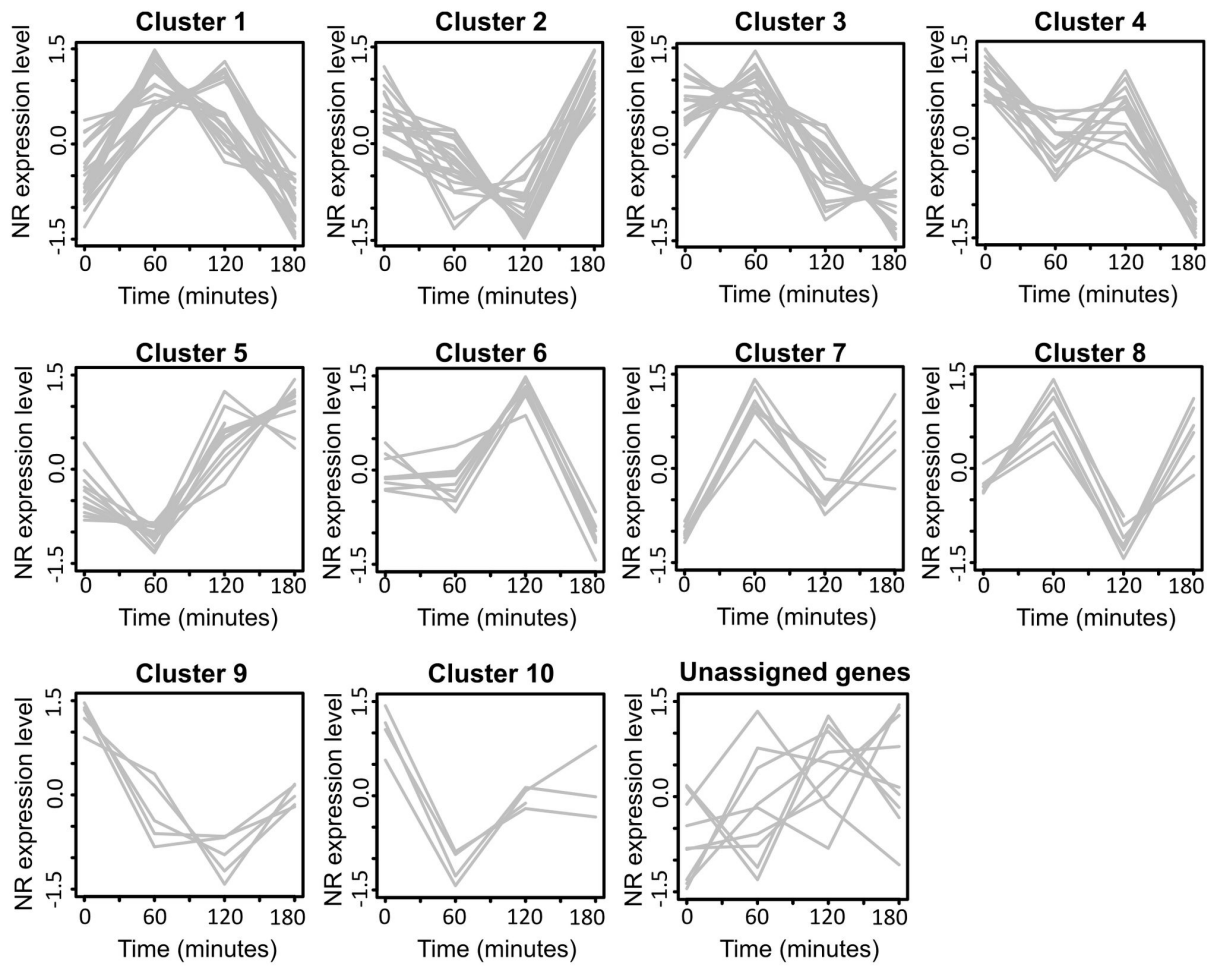
TF and TR genes co-expressed under conditions of low CO_2_ concentration. The analysis of the TF and TR transcript levels revealed co-expressed genes, clustered according to similar expression profiles using the quality threshold method with Pearson correlation as similarity measure. The y axis shows normalized expression levels as relative expression values (log_2_), scaled by gene minima and maxima and mean centered. At time zero (0 min) the carbon dioxide concentration in the growth medium was shifted from 5% to 0.04%. Samples were taken immediately before the shift (indicated as 0 min) and after 60, 120 and 180 min. Sampling time points are shown on the x axis.

We used reverse transcription-quantitative PCR (RT-qPCR) to monitor changes in transcript abundance of 165 *C. reinhardtii* TF and TR genes. We detected transcripts of 130 genes and these were monitored at four different experimental time points, i.e., 0, 60, 120 and 180 min, after the HC-to-LC shift. We could not detect any expression for 35 genes, either because their transcript abundance was below the RT-qPCR detection limit or because the respective genes were not expressed in the algal cells under our culture conditions. Co-expressed genes were identified and clustered in groups showing similar expression profiles (Figure **5**) using the quality threshold (QT) clustering method [23]. Several TF and TR genes were differentially regulated upon the decrease in CO_2_ concentration; 121 genes were grouped into ten clusters whilst nine genes could not be assigned to any cluster (see Material and Methods, section Reverse transcription - quantitative PCR data analysis). A list of genes and their respective clusters are presented in Table **S2**. Genes induced or repressed by more than 2-fold are listed in Table **S3**. From the set of genes regulated after the HC-to-LC transition, the expression of 21 genes changed by at least 2-fold already within one hour after the CO_2_ concentration shift, including the following (referenced by their TF/TR family name; Table **S3**): DDT (protein ID 8155; fold-change FC > 8), CSD (protein ID 126810; FC > 6), MYB-related TF (protein ID 166618; FC > 5), HSF (protein ID 117914; FC > 4), and SNF2 (protein ID 111277; FC > 3). Our experimental results showed that *Lcr1* (protein ID 184359) was among the five most strongly expressed genes in cells cultivated at LC condition for 180 min. Other TF and TR encoding genes were also expressed at high levels. However, to our knowledge, only *Lcr1* has been functionally characterized with respect to CCM.

### Reconstruction of the gene regulatory network underlying CCM induction

**Figure 6:**
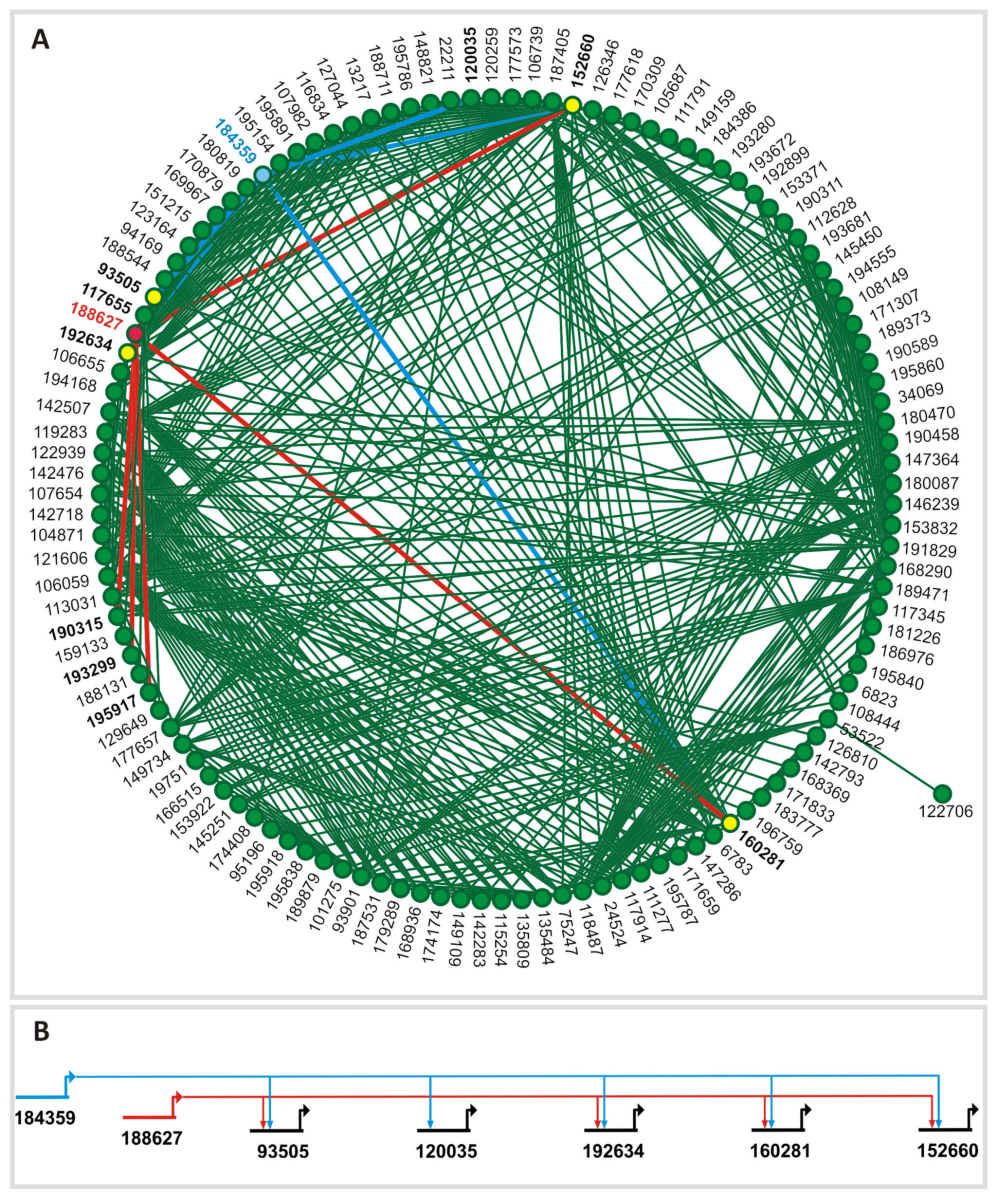
Graph representation of the reconstructed directed gene regulatory network of *Chlamydomonas* grown in low-CO_2_ condition. A directed GRN of *C. reinhardtii* was reconstructed using the IOTA method. (A) Graphical representation of the topology of the whole network, generated using Cytoscape (http://www.cytoscape.org). The numbers indicated in the figure correspond to the protein IDs of the corresponding genes (nodes). The interacting partners of the two MYB-related TFs that are highly expressed in LC condition are highlighted. One of these genes encodes for the Low-CO_2_ response regulator 1 (*Lcr1*, protein ID 184359; blue node) and when compared to the other MYB-related TF (*Lcr2*; protein ID 188627; red node) shows common interacting partners (yellow nodes). Blue and red edges link *Lcr1* and *Lcr2* to the genes they may regulate. (B) Set of genes predicted to be regulated by *Lcr1* (184359, blue lines) alone or by both, *Lcr1* and *Lcr2* (188627, red lines) are shown in detail and the directionality of the regulatory event is indicated by the direction of the arrows. The model was generated using BioTapestry (http://www.biotapestry.org/).

We used the recently established IOTA method [19] to reconstruct the GRN of *C. reinhardtii* cells cultured in LC condition. The reverse-engineered topology of the directed network revealed regulatory links of 122 TF and TR coding genes underlying the CCM regulatory network (Figure **6A**; Figure **S2**, File **S1**, File **S2** and File **S3**). This network includes previously known LC-responsive regulators such as *Lcr1* (Figure **6B**) and generated new insights into the regulation of the CCM. On average, each TF or TR from the reconstructed GRN regulates four other genes. A matrix containing all interaction pairs obtained with the IOTA method together with their probability values is given in Table **S4** (data are also available through our web site at http://plntfdb.bio.uni-potsdam.de/ChlamyTRI/). 

## Discussion

### Identification of putative regulatory elements of the Cah1 gene

Many genes have previously been shown to be up-regulated in *C. reinhardtii* cells during CCM induction. Among them is *Cah1*, which encodes periplasmic carbonic anhydrase (chromosome_4:1849052-1853308, protein ID 24120; name: estExt_fgenesh1_pm.C_150006, *Chlamydomonas* genome annotation v.4) [13]. The 5' upstream region of the *Cah1* gene contains at least one silencer and one enhancer region [20]. Two putative enhancer elements (EEs), i.e., EE-1 (AGATTTTCACCGGTTGGAAGGAGGT; -293 bp to -269 bp upstream of the translation initiation codon) and EE-2 (CGACTTACGAA; -241 bp to -231 bp upstream) were previously found in the enhancer region [20]. Earlier experimental findings indicated the presence of a nuclear protein that binds to the two EEs independent of CO_2_ concentration and light. Within the two EEs, a sequence motif (consensus sequence 5´-GANTTNC-3´) was previously found [24]. This motif shows high sequence similarity to the plant box III motif which has been shown to be a light-responsive element. Our findings confirmed the presence of a nucleosome depleted region within the *Cah1* promoter, in agreement with the previously identified enhancer elements in the same *Cah1* promoter segment [20]. We also detected nucleosome depletion in the 3´ UTR of the *Cah1* gene; however, the functional significance of this observation remains to be determined.

There is evidence showing that expression of carbonic anhydrases (CAs) is regulated by transcription factors that are responsive to environmental changes in [CO_2_] [8,15,25]. Studies using transcript quantification technologies such as microarrays and RNA-seq revealed the expression profiles of CAs in *Chlamydomonas* under LC condition and light [8,15,25,26]. The RNA-seq analysis revealed that not all CAs are overexpressed under LC condition [26]. Furthermore, comparing results from different studies we observed that only three of the twelve annotated CAs in *Chlamydomonas* are overexpressed (>2-fold change) under LC [8,10,25,26]. *Cah1*, *Cah4* and *Cah5* transcripts were up-regulated under LC condition in RNA-seq data from wild-type strains cc124 and cc125 [25,26]. The other CAs were down-regulated, not induced or slightly modulated (<2-fold expression change), indicating that a complex gene expression regulatory network may control the expression of these enzymes. Our FAIRE-seq experiment revealed FAIRE summits both, at the *Cah1* locus, chromosome_4:1849052-1853308 (FAIRE summits 5´ up- and 3´ downstream of the annotated transcript), and the *Cah4* locus, chromosome_5:3320850-3322660 (FAIRE summit 3´ downstream of the annotated transcript). No FAIRE summit was found within a region of 1 kb up- or downstream of the *Cah5* annotated transcript (chromosome 5:3326106-3327036), or in its introns or exons. Of note, the *Cah5* gene is in close proximity to the *Cah4* gene. With respect to CAs weakly or not induced under LC we could track the genomic coordinates of five genes (*Cah2*, *Cah3*, *Cah6*, *Cah7* and *Cah8*) in our LC FAIRE-seq data. We did not observe FAIRE summits in the proximity (up to 1 kb up- and downstream) of the *Cah2* (chromosome_4:1844172) and *Cah3* (chromosome_9:4490493-4492561) transcripts; we found FAIRE summits in the first exon of the *Cah6* (chromosome_12:210284-213171) gene, in the first intron of the *Cah7* gene (chromosome_13:64820022-6487131), and only *Cah8* (chromosome_9:3001516-3007085) had a FAIRE summit at the promoter region (all data can be observed in detail through our FAIRE browser). These results indicate that the presence of a FAIRE summit in a promoter region of a specific gene may indeed indicate gene expression modulation; the absence of a FAIRE summit or its presence in introns or exons may indicate low induction or no expression of a specific gene. It is also likely that different regulatory networks are responsible for the control of the cellular responses in different acclimation states under low, very low or high CO_2_ concentration. However, there is currently no complete picture available that could explain the variations in gene expression regulation of CAs in response to different CO_2_ concentrations.

### FAIRE-seq and motif analysis revealed the genome-wide location of putative regulatory elements and co-regulated genes

The FAIRE-seq approach revealed the identity of nucleosome-depleted regions and potential genomic regulatory elements. We detected FAIRE peaks close to coding regions of seventeen of the TF and TR genes analyzed by transcript profiling. We reasoned that these regulatory genes may share similar sequence motifs in their promoters. To investigate this possibility, we analyzed their 5´ upstream regions using MEME, which revealed the presence of statistically significant motifs (Table **S5**). Ten motifs identified by MEME were also analyzed using the MAST tool to determine their specificity and statistical significance compared to motifs found in a global set of 14,598 promoter sequences extracted from the *Chlamydomonas* genome annotation v.3.1 (DOE/JGI). MAST analysis revealed that the combination of motifs found in the promoters of the seventeen TF and TR genes for which corresponding FAIRE peaks were detected is significantly overrepresented (*p* ≤ 0.0001) compared to the whole promoter dataset, indicating gene co-expression. A motif analysis was also performed with the whole set of FAIRE peaks detected. Regions ±100 bp up- and downstream of the FAIRE summits were analyzed using the MEME tool, revealing the presence of conserved overrepresented motifs along the genome (*p* ≤ 0.0001). A list of these sequence motifs is given in Table **S6**. 

### Early responsive TF and TR genes may be involved in chromatin remodeling

Under CO_2_ limitation, cellular pathways trigger the induction or repression of genes necessary for the establishment and control of the CCM [14]. Among the genes responding within one hour of the HC-to-LC shift are candidate chromatin remodeling proteins. The TR DDT was predicted to have three domains, i.e., bromodomain, DDT and PHD. So far, only one *DTT* gene was identified in the *C. reinhardtii* genome [17]. Bromodomains are important for chromatin remodeling recognizing acetylated lysines on core histones and promoting nucleosomal disassembly [27]. Histone acetylation directly correlates with increased RNA synthesis rates [28]. Currently, however, in many cases it is not fully understood how histone modifications and the interplay with cofactors regulate the transcription of a given gene *in vivo* [29,30].

A further early responsive transcription regulator gene identified encodes a member of the cold shock domain (CSD) protein family that harbors two domains, CSD and an RNA-recognition motif (RRM) [31]. In contrast to prokaryotic CSD proteins, eukaryotic proteins containing a CSD may also contain other domains and their functions are not exclusively related to cold shock responses [32]. A major cold shock protein in *Arabidopsis thaliana*, CsdP1, which contains a CSD domain, has been shown to exhibit RNA chaperone activity [33,34]. In our experiment, expression of the *Csd* gene (protein ID 126810) was up-regulated within one hour after the HC-to-LC shift and started to decrease after two hours. It may therefore be hypothesized that the proposed RNA chaperone function of Csd is important during the initial phase of the CO_2_ concentration shift to favor translation of newly formed transcripts.

The MYB-related TF early induced under LC condition (protein ID 166618) contains a single MYB DNA-binding domain. Its transcript abundance was previously reported to increase upon CO_2_ deprivation, but the biological function of the encoded TF has not yet been characterized [35,36]. In *C. reinhardtii*, the MYB domain-containing transcription factor Psr1, a member of the G2-like TF family, regulates phosphate uptake. Mutants lacking a functional *Psr1* gene are impaired in the proper acclimation of the cells to phosphorus deficiency [37]. 

Our data also revealed up-regulation of *Hsf1* (protein ID 117914), a member of the heat shock transcription factor (HSF) family. *C. reinhardtii* has two annotated HSF genes (*Hsf1* and *Hsf2*), while *A. thaliana* has at least 21 genes. Notably, in the absence of heat stress, HSF proteins may bind to heat shock elements in the promoters of target genes thereby driving basal transcriptional activity [38]. Previous studies revealed enhanced expression of *C. reinhardtii Hsf1* after heat shock. RNA interference (RNAi) lines generated for *Hsf1* showed reduced expression of *Hsf2* and *Hsp90C* during heat shock and it has been suggested that Hsf1 constitutively and specifically interacts with Hsp70A and that it could be a key regulator in stress responses in *C. reinhardtii* [39,40]. The over-expression of *Hsf1* during the initial CCM induction phase could also be regarded as additional evidence for a role of HSF proteins in regulating gene transcription, likely recruiting remodeling factors which may together function in the transient displacement of histone octamers [41]. It has recently been reported that Hsf1 binds to promoter elements of the target genes *HSP22F* and *HSP70A* where it plays a role in the acetylation of histone H3 and H4 located on these promoters, which may be a prerequisite for the transcription of such genes [40]. 

Another gene highly expressed shortly after the HC-to-LC shift encodes a TR protein of the SNF2 family (protein ID 111277); this gene shows high sequence similarity (e-value = 5e-174; DNA level) to *Rad54* from *Arabidopsis*. Rad54 is involved in chromatin remodeling [42], has an important role in homologous recombination [43], and enhances resistance to DNA damage when over-expressed [44]. In the future it will be important to study mutants of these early responsive transcription control genes to unravel their role for CCM establishment in *C. reinhardtii*. 

### Reconstructed CCM gene regulatory network reveals new candidate target genes

We used IOTA (inner composition alignment) [19] to reconstruct the GRN underlying the CCM and through this approach were able to predict new regulatory links. For example, the reconstructed GRN predicts that *Lcr1* regulates five other TF or TR genes (protein IDs 93505, 120035, 152660, 160281 and 192634) (Figure **6B**). We identified two additional genes (a MYB-related TF, protein ID 188627, and an SNF2 family TR, protein ID 188544) that may regulate a common subgroup of genes predicted to be regulated by *Lcr1*. 

The MYB-related TF (protein ID 188627), from now on *Lcr2* for *Low-CO*_*2*_ *response regulator 2*, was predicted to regulate four (protein IDs 93505, 152660, 160281 and 192634) of the five genes putatively regulated by *Lcr1* (Figure **6B**). The four genes are members of the following TF and TR families: SBP, Orphan, FHA and C3H. A motif search using MEME revealed similar sequence motifs and hence possible *cis*-elements in the promoter regions of *Lcr1* (protein ID 184359) and *Lcr2* (protein ID 188627) (Table **S7**). This finding suggests that both genes may be co-regulated by similar upstream DNA-binding proteins. Not surprising, not all motifs found were specific to these two promoters. When all motifs were analyzed individually using the MAST tool and compared against the promoter sequences of the 14,598 *C. reinhardtii* genes, some were not significantly overrepresented (*p*-value > 0.0001). However, Motif 4 (Table **S7**) appeared to be highly specific for the *Lcr1* and *Lcr2* promoters, which according to the MAST analysis were the only ones containing it (*p*-value < 0.0001), reinforcing the model of co-regulation of both TFs during carbon deprivation. 

The SNF2 TR (protein ID 188544) was predicted to regulate three (protein IDs 93505, 152660 and 192634) of the five genes downstream of Lcr1. Our transcript profiling revealed that the transcription of these putative target genes is repressed after three hours at LC condition (see Table **S3**), indicating a possible role of Lcr1 as well as Lcr2 and the SNF2 TR (188544) as repressors of CCM-related genes. These results provide new insights into the regulation of CCM-related genes, revealing novel candidate regulatory genes. 

In conclusion, the cellular response of *C. reinhardtii* to reduced external availability of CO_2_ involves the transcriptional reprogramming of genes crucial for CCM regulation. It has been demonstrated previously that upon CO_2_ deprivation, *C. reinhardtii* modulates the expression of genes related to carbon uptake, photosynthesis and photorespiration. However, so far there has been no comprehensive study of expression changes of transcriptional regulators or the genome-wide location of the regulatory elements involved in the CCM. In our present study, we performed a genome-wide analysis, using FAIRE-seq, of the nucleosome occupancy of cells cultured for three hours in LC condition. We identified another low-CO_2_ responsive gene, *Lcr2*, the promoter of which contains sequence motifs similar to those found in the *Lcr1* promoter, suggesting that both genes may be regulated by the same or similar upstream DNA-binding protein(s) domains ensuring co-regulation. Furthermore, we predicted the GRN for CCM which revealed the interaction of more than 100 genes and will serve as a basis for selecting further candidate genes and related sub-networks. We propose that combining gene expression profiling, genome-wide mapping of nucleosome-depleted chromatin regions by e.g. FAIRE-seq, and mathematical reverse-engineering of network topology accelerates the discovery of gene regulatory networks. Our analysis identified several new candidate genes of potential relevance for the carbon deprivation response and the genomic coordinates of nucleosome depleted regions, which may function as regulatory elements important for CCM; their role can now be studied in detail using available molecular and genetic tools. 

## Materials and Methods

### Cell growth conditions and strain

*C. reinhardtii* (strain cc503 cw92 mt+) was cultured in 2-L flasks in photoautotrophic and temperature-controlled condition. Cells were cultured in H_20_P medium (modified TAP medium without acetate, supplemented with 20 mM HEPES as buffering agent, replacing Tris) under continuous light (~200 µE m^-2^ s^-1^) and constant supply of air with 5% CO_2_ (gas mixture was provided by a B-DCU Biostat fermenter, Sartorius, Göttingen, Germany, and controlled via a GC system, Agilent Technologies 3000 micro-GC, Waldbronn, Germany). After the cell culture had reached an optical density of 0.5 at 750 nm (approximately 3x10^6^ cells · mL^-1^), 900 mL were sampled for e.g. transcript profiling, and the concentration of CO_2_ in the remaining culture was reduced to 0.04% in air. Samples of 20 mL of cell culture were then collected after 60 min and 120 min for transcript profiling, and 1 L of culture was sampled after 180 min for transcript profiling and FAIRE. Cells harvested for transcript analysis were collected by centrifugation (in 2-mL aliquots) at 3,000 *g* for 2 min and the cell pellets were then immediately frozen in liquid nitrogen. The samples were kept at -80°C until further use. Cells (900 mL) from the last time point (180 min) were directly subjected to formaldehyde crosslinking for FAIRE (see description below). 

### Design of primers for RT-qPCR and validation

The design of primer pairs was based on the *C. reinhardtii* gene models of the genome annotation v.3.1 released by the Joint Genome Institute (DOE/JGI; http://genome.jgi-psf.org/Chlre3/Chlre3.home.html). Primer pairs were designed for the predicted transcription factor and transcription regulator genes reported in the Plant Transcription Factor Database [17]. Primers for the carbonic anhydrase encoding gene *Cah1* (GI:159468241, GeneBank at NCBI) were designed based on the coding DNA sequence (genomic coordinates: chromosome_4: 1849052.1853308) obtained from the *C. reinhardtii* genome annotation v.4 (http://genome.jgi-psf.org/Chlre4/Chlre4.home.html). Primers for the mitochondrial carbonic anhydrase gene *Mca* (GI: 8287171) were designed based on the coding DNA sequence (coordinates: scaffold_289:8821-10643) of the *C. reinhardtii* genome annotation v.3 (http://genome.jgi-psf.org/Chlre3/Chlre3.home.html). Criteria for primer design were as follows [45]: Tm = 60 ± 1°C, length 18 to 25 bases, preferentially on exon-exon junctions. When possible, primers were designed to have a GC content of 45 to 55%, generating a single PCR product sizing between 60 and 150 bp. Primers were synthesized by Eurofins MWG Operon (Ebersberg, Germany). A complementary *in silico* validation of the primer specificity was carried out using the computational tool QuantPrime [46]. Experimental validation of primer pairs was performed by checking the presence and approximate size of PCR products obtained from the amplification of cDNA synthesized from RNA extracted from *C. reinhardtii* cells grown under photoautotrophic conditions. PCR products were separated in 3% agarose gels and amplicons which did not have the correct size (i.e., if they deviated by more than ~20 bp from the expected size) were excluded from further analysis. A list with the sequences and respective identifiers of the primer pairs is given in Table **S8**.

### RNA extraction, cDNA synthesis and RT-qPCR

A reverse transcription-quantitative PCR (RT-qPCR) approach was used for the analysis of the transcript levels of TF and TR encoding genes, *Actin*, *Cah1* and *Mca*. Total RNA extraction was performed using the RNeasy Plant Mini Kit (Qiagen, Hilden, Germany). Briefly, frozen cell pellet originating from 2 mL culture was resuspended in 450 µL lysis buffer RLT and frozen again in liquid nitrogen for 3 min. Cells were then lysed by incubating them for 3 min at 56°C. Further steps of the RNA extraction procedure were performed as described by the manufacturer. To remove genomic DNA from the RNA extracts, DNA was digested on-column using DNAse I (Qiagen); the isolated RNA was then subjected to an additional DNAse treatment using TURBO DNase (Ambion, Darmstadt, Germany) as indicated by the manufacturer. The integrity of the RNA was checked by electrophoresis on 2% denaturing agarose gels; RNA quality was assessed by determining the 260 nm/280 nm absorbance ratio using a Nanodrop ND-1000 instrument (Thermo Scientific, Schwerte, Germany). Furthermore, the absence of genomic DNA in the RNA extracts was assessed by performing a qPCR on a 1-µL aliquot of each sample of total RNA using a pair of primers annealing to an intergenic region of chromosome 16 (forward primer 5’-TGTCTTGTGAATCCTGCCCTC-3’ and reverse primer 5’-AAAGAGCTCACAAGTACACACCGA-3’). Only when the qPCR analysis of the RNA sample resulted in a C_t_ value greater than 36 the RNA was used for cDNA synthesis. Three micrograms of total RNA were used for cDNA synthesis employing the SuperScript III First Strand System (Invitrogen, Darmstadt, Germany) according to the manufacturer’s instructions, using oligo-(dT_20_) as primer for the synthesis of the first complementary DNA strand. cDNA synthesis efficiency was estimated by RT-qPCR using primer pairs for the ubiquitin protein ligase encoding gene (protein ID 190824; forward primer 5’-TTACCTGCCTTCCGATTGCGTAGC-3’ and reverse primer 5’-TTACTATGCCTGAGCACGCAGCAC-3’). The cDNA samples were diluted ten times prior to the final PCR reaction which was conducted with SYBR Green mix (Applied Biosystems, Darmstadt, Germany) in a final reaction volume of 5 µL containing 5 µM primers. Dilution of primers and pipetting into the PCR 384-well plates were performed with a robot to increase accuracy and throughput. The ABI PRISM 7900HT sequence detection system (Applied Biosystems) was used for the RT-qPCR reactions which were carried out as previously described [47].

### Analysis of reverse transcription - quantitative PCR data

Raw RT-qPCR data were pre-processed with the program SDS v.2.3 (Applied Biosystems) and the amplification curves were analyzed considering a threshold of 0.2 for the variation of the fluorescence of the sample to the fluorescence of the passive dye to obtain the C_t_ values (cycle threshold). The baseline measurement was taken from the 3^rd^ to the 15^th^ cycle. Quality controls were done in order to evaluate the raw data by means of efficiency of the amplification reactions using log-linear regression as described previously [48] and the patterns of the melting curves. Genes for which the amplification efficiency was less than 95% in more than 25% of the reactions were excluded from further analysis. Reactions presenting multiple melting peaks or melting temperatures which disagreed with the expected temperature were also excluded from further analysis. Only high-quality measurements were selected for relative gene expression analysis. All raw C_t_ values of TF and TR genes were normalized by the Quantile method using the R package qpcrNorm [49], available through the Bioconductor project (www.bioconductor.org). Biological medians of the normalized C_t_ values (ΔC_t_) from the five biological replicates were calculated. The relative gene expression levels were calculated as delta-delta C_t_ values (ΔΔC_t_ = ΔC_t LC_ - ΔC_t HC_). Fold change (FC) was calculated as 2^-ΔΔCt^ [45]. Expression values were normalized by genes and mean centered before the clustering analysis, performed using the MeV suite v4.6. (TM4 Microarray Software Suite) [50]. Clustering of the gene expression profiles was performed using the Quality Threshold (QT) algorithm (minimum cluster size of 4 genes, diameter 0.5, and Pearson correlation as similarity measure) [23]. The QT method ensures that genes will not be included in a cluster if they exceed the defined quality diameter threshold. Expression levels of *Cah1* and *Mca* were determined from three experimental replicates and expression levels calculated by the ΔΔC_t_ method as previously described [47] using the actin encoding gene (GI:159482013; forward primer: 5´-GCGGCTAACGACGGAGGAT-3´; reverse primer: 5´-CCATGACCCGCTCCTCATATC-3´) as a reference for normalization.

### Formaldehyde-assisted isolation of regulatory elements (FAIRE)

We used the FAIRE method [51,52] for the identification of nucleosome-depleted chromatin regions likely containing regulatory elements. FAIRE was previously used for studies in e.g. yeast [22], human cells [51] and maize (*Zea mays*) [53]. However, genome-wide FAIRE patterns have to our knowledge not yet been reported for higher plants or algae. 

For the isolation of regulatory elements by FAIRE, 900 mL of *C. reinhardtii* cell culture was sampled at time zero (cell culture at 5% CO_2_) and three hours after the shift from HC to LC and crosslinked by stirring at 150 rpm for 5 min at room temperature with 100 mL of 10 x crosslinking buffer (10% formaldehyde, 100 mM KCl, 100 mM NaCl, 100 mM HEPES, pH 8.0) reaching the final concentration of 1% formaldehyde. The crosslinking reaction was quenched by adding 1 M glycine to a final concentration of 125 mM and stirring at 150 rpm for 5 min at room temperature. Cells were harvested by centrifugation at 22°C for 3 min at 3,000 *g* in 1-L bottles using a Beckman coulter Avanti J series centrifuge with fixed-angle rotor model JLA-16.250 (Beckman Coulter, Krefeld, Germany). The cell pellet was suspended in 10 mL of resuspension buffer (0.5% Triton X-100, 10 mM HEPES, 0.5 mM EDTA, 10 mM KCl, 10 mM NaCl), transferred to 50-mL conic tubes and centrifuged at 4°C for 3 min at 3,000 *g*. The pellet was resuspended with 10 mL of lysis buffer (1% Triton X-100, 10 mM KCl, 1.5 mM MgCl_2_.6H_2_O, 0.2 M sucrose, 10 mM HEPES, pH 8.0) supplemented with 1:100 plant protease inhibitor cocktail (Sigma, #P9599) and fresh 1 mM DTT. The samples were incubated for 10 min on ice and centrifuged at 4°C for 20 min at 1,000 *g*. The lysis step was repeated once and the final pellet was suspended in 2 mL of nuclear lysis buffer (140 mM NaCl, 1 mM EDTA, 1% Triton X-100, 0.1% sodium deoxycholate, 1% sodium dodecylsulfate, 50 mM HEPES, pH 8.0) supplemented with 1:100 plant protease inhibitor cocktail (Sigma, #P9599) and fresh 1 mM DTT. An aliquot of 500 µL was separated to further check chromatin crosslinking efficiency. Genomic DNA crosslinked with formaldehyde to proteins and de-crosslinked thereafter (but not fragmented) was easily recovered from the aqueous phase after phenol-chloroform extraction (Figure **S3A**, lane 1). On the contrary, unsheared genomic DNA crosslinked to proteins aggregated in the interphase between the aqueous and organic phases, as expected, and was therefore not detected in the aqueous fraction by gel electrophoresis (Figure **S3A**, lane 2). We observed efficient DNA-protein crosslinking in cells treated with 1% formaldehyde (Figure **S3B**, lanes 2-4); de-crosslinked fragments were easily recovered from the aqueous phase after phenol-chloroform extraction, with maximal yield from cells previously crosslinked for 5 min (Figure **S3C**, lane 4). The verification of crosslinking efficiency is important to assure that only nucleosome-depleted regions of the chromatin are isolated. Only samples with maximal crosslinking should be further subjected to the purification steps. 

Samples were sonicated on ice (5 cycles at 40% power, 9 cycles at 10% power, 10 sec/cycle) using a Sonopuls HD 2070 sonicator (Bandelin Electronic, Berlin, Germany). After sonication, samples were centrifuged at 4°C at 4,000 *g* for 5 min. The supernatant was kept at -80°C until further use. An aliquot of 500 µL from the extracted sonicated chromatin was taken for gel analysis (3 µL) and to serve as a control sample. 

Control samples were de-crosslinked by overnight incubation at 65°C. Both, control and sonicated chromatin were subjected to a standard nucleic acid extraction procedure using phenol:chloroform:isoamylalcohol (Roth, Karlsruhe, Germany) as previously described [51] and the samples containing the FAIRE-extracted DNA fragments were kept at -80°C until further analysis.

### Quantitative PCR analysis of FAIRE-isolated DNA fragments

Quantitative PCR was used to check for enrichment of defined DNA segments in the FAIRE samples. We selected the *Cah1* genomic locus (chromosome_4:1849052-1853308, protein ID 24120; name: estExt_fgenesh1_pm.C_150006, *Chlamydomonas* genome annotation v.4) as a marker. The selected genomic fragments encompass coding and non-coding regions (including the 5´ upstream sequence). In *C. reinhardtii*, *Cah1* transcription increases upon CO_2_ limitation. Previous studies revealed the position of putative enhancer and silencer elements in the promoter region 5´ upstream of the translation initiation codon (ATG) of *Cah1* [24]. Primer pairs (Table **1**) were designed for ten segments of the genomic *Cah1* locus using QuantPrime [46]. Figure **1** shows the regions covered by the primer pairs. FAIRE samples were diluted to a final concentration of 50 ng DNA µL^-1^ before running the qPCR reactions and 1 µL was mixed with 5 µL of 2x SYBR Green mix (Applied Biosystems) to a final reaction volume of 10 µL containing 5 µM primers. The reactions were conducted in 384-well plates and PCR amplification was conducted on an ABI PRISM 7900HT sequence detection system (Applied Biosystems) as previously described [47]. To calculate the enrichment of genomic regions we used a method previously described [51]. Briefly, the averages of the resulting C_t_ values for the control and FAIRE samples were calculated. The ratio between FAIRE to control samples (FAIRE/control) was determined for each segment of the *Cah1* gene. This analysis was performed for samples harvested before the shift from HC to LC and for samples harvested three hours after the shift. For each sample the average C_t_ value of the nearest region of the segment having the lowest resulting ratio (FAIRE/control) was taken as a reference region for calculating the ΔC_t_ values (C_t region of interest_ - C_t reference region_). The ΔΔC_t_ values (ΔC_t FAIRE sample_ - ΔC_t control sample_) were calculated and the enrichment level determined as 2^-ΔΔCt^. FAIRE experiments were done in triplicate and three technical replicates were done for each segment analyzed by qPCR.

**Table 1:** Primer pairs used for enrichment analysis of FAIRE fragments from the *Cah1* locus through quantitative PCR.

	**Primer pair coordinates** *	**Sequences of oligonucleotides**		
Amplicon identifier**	Start/end		Forward primer (5´-> 3´)	Reverse primer (5´-> 3´)	
A	660/729		TAGCCTTTCAAGCCGCGCCA	GCGTCAAACCTCCTTCCAACCG	
B	804/867		GCCGGAACTCCAACCAAGTAGC	GCATTTCTGCATGCGCACAGT	
C	937/1000		CCGCCGTACCGTTGTCACTTT	CGCAGGGTGTGGACAACTATGG	
D	1574/1637		TAGGGCGATGAACGGTTCTGGT	TCCTTGCCCTCCCTGTATGTGG	
E	2485/2545		TGGAGTGCGAGCACGCTTAGTA	CCAAGGCACCCAGATGCATGAC	
F	2940/3008		GTTCCACTTCCACTCCACCTCG	AAAGTCAAGAGTTGCGCCCACG	
G	3602/3663		CATCAAGCTGGGTGAGCTGCTG	TGAGGCTGCCCTCGTACGTTA	
H	3719/3781		CATCAGCTTCGGCCAGTGGAAC	GTGGAGTTGCACTCCTTCAGGC	
I	4714/4773		ATGGAGTTGGTTCCACGATGGG	ACGTCCGGCCCACTGACTTTAT	
J	4996/5062		CTGTCTGGCTGGCTGGGTTGTT	TGCATTGATCGGCATGTGCGAC	

* Coordinates relative to the *Cah1* locus (chromosome_4:1849052-1853308).

** The positions of the amplicons along the *Cah1* locus are shown in Figure **1**.

### Deep sequencing

For the genome-wide identification of nucleosome-depleted regions and putative regulatory elements of *C. reinhardtii*, DNA fragments present in the FAIRE samples were sequenced by deep sequencing technology. So far, no FAIRE-seq data were published for plants and no protocol was available for such an analysis in *C. reinhardtii*. A FAIRE sample from one of the three experimental replicates analyzed previously by qPCR was selected for deep sequencing. The chromatin was extracted from cells cultured for 3 h under LC condition. After isolation of nucleosome-depleted genomic fragments by FAIRE the DNA sample was fractionated on a 2% agarose gel and fragments between 200 to 400 bp were used for the construction of a DNA library for sequencing. DNA library preparation and DNA sequencing were performed by LGC Genomics (Berlin, Germany) using Paired-End-DNA Sample Prep Kit, HiSeq Paired-End Cluster Generation Kit with paired-end flow cells, and TruSeq SBS Kit HS (200 cycles) according to the user guide instructions (Illumina, Eindhoven, The Netherlands). A total of 350 ng of double-strand DNA containing the FAIRE fragments was subjected to end-repair and adenylation of the 3´ ends for ligation of adaptors. The ligation products were purified from a gel and the fragments amplified by PCR (12 cycles). The final PCR product was purified with Ampure beads (Beckman Coulter Genomics, Bernried, Germany) and the library validated and quantified using a Bioanalyzer (Agilent, Waldbronn, Germany) and Nanodrop ND-1000. Amplified DNA fragments were sequenced with a HiSeq2000 sequencing system (Illumina) and all raw data were submitted to GenBank and are deposited in the Sequence Read Archive (SRA) of the European Nucleotide Archive (ENA) under the accession number ERP001835 (http://www.ebi.ac.uk/ena/data/view/ERP001835). Paired-end sequence reads of 50 bp were mapped against the masked *Chlamydomonas* genome sequence v.4.0 (DOE/JGI Joint Genome Institute; http://genome.jgi-psf.org/Chlre4/Chlre4.home.html) using BowTie [54], allowing two-nucleotide mismatches at maximum for each DNA read. Mapping files on SAM format were manipulated with SAMtools (http://samtools.sourceforge.net/) and BEDtools (http://code.google.com/p/bedtools/) in order to export mapping positions into BED format. FAIRE-seq peaks were identified using MACS tool v. 1.4.0beta [21] with a *p*-value cutoff equal to 10^-5^, and mapped to the *Chlamydomonas* genome annotation v. 4.0 (DOE/JGI) to find the closest annotated features, namely genes.

### Reconstruction of gene regulatory networks (GRNs)

To identify regulators of *C. reinhardtii* during CCM, we used the IOTA (inner composition alignment) method [19,55], a permutation-based measure recently developed for the detection of regulatory links from very short time series typically obtained from high-throughput experiments [19,55]. We applied IOTA to the expression data of TF and TR genes analyzed in the present study in order to reconstruct the topology of the underlying regulatory network. Furthermore, we performed a statistical analysis based on the following permutation test: we selected 10,000 permutations uniformly at random, shuffled the data according to these permutations, recalculated IOTA and estimated the empirical *p*-values at significance level 0.01.

### Motif analysis

To test whether the promoter regions of genes of interest had common sequence features, 5´ upstream regions of selected genes were retrieved from the *Chlamydomonas* genome annotation v.3.1 (DOE/JGI) and overrepresented sequence motifs were identified using the MEME suite (v.4.3.0, http://meme.nbcr.net/) [56], allowing a maximum of 10 motifs with a maximum length of 20 nucleotides to be reported. Other parameters were used as default settings. When significant motifs were found, an analysis of their specificity was conducted using MAST (http://meme.sdsc.edu/meme4_3_0/cgi-bin/mast.cgi) and a file containing 14,598 promoter sequences obtained from the *Chlamydomonas* genome annotation v.3.1 (DOE/JGI). Additionally, a motif search was performed to identify conserved sequences in regions that included sequences up- and downstream (± 100 bp) of the FAIRE summits. A fifth-order Markov model was applied as a background for the motif search using the MEME suite, allowing a maximum of 10 motifs with a maximum length of 50 nucleotides to be reported.

## Supporting Information

Table S1 **Coordinates of FAIRE peaks found close to TF and TR genes regulated by low CO_2_.** Excel file listing the genomic positions of the FAIRE peaks found close to a TF or TR gene transcript. Protein ID, transcript ID, gene family name, peak name, peak coordinate and peak score are given.
(XLS)

Table S2 **CCM TF and TR genes and clusters of expression profiles.** Excel file listing the genes assigned to different clusters of gene expression profiles. Protein ID, transcript ID, gene family name and cluster ID are given.
(XLS)

Table S3 **TF and TR genes showing an expression fold change larger than 2.** Excel file listing TF and TR genes showing an expression fold change larger than 2 in at least one experimental time point. Protein ID, transcript ID, gene family name, cluster ID and gene expression fold change (FC) for time points corresponding to 60, 120 and 180 min after the shift from HC to LC condition are given. Expression values ± SD from five biological replicates are shown.
(XLS)

Table S4 **Reconstructed gene regulatory network of *Chlamydomonas* under low-CO_2_ (LC) condition.** Excel file containing the probability matrix for the interacting genes used in the reconstruction of the gene regulatory network of CCM. Data are also available from the *Chlamydomonas* Transcriptional Regulation Initiative (Chlamy TRI) web portal: File URL: http://plntfdb.bio.uni-potsdam.de/ChlamyTRI/. Registration at the website is necessary to retrieve the data set.
(XLS)

Table S5 **Motifs found in promoter regions of genes regulated by low CO_2_ and having a close FAIRE peak.** Excel file listing the sequences of the motifs found in the promoter regions of genes regulated by low CO_2_ and having a close FAIRE peak. Protein ID, transcript ID, gene family name, motif identifiers and motif sequences are provided.
(XLS)

Table S6 **Motifs found in sequences neighboring FAIRE summits.** Excel file listing the motifs identified in DNA sequences located up- or downstream (±100 bp) of the FAIRE summits, identified by FAIRE-seq analysis. Motif ID, transcript ID, motif identifiers and sequences are provided.
(XLSX)

Table S7 **Sequence motifs found in the *Lcr1* and *Lcr2* promoters.** Excel file listing the sequences of the motifs found in the promoter regions of *Lcr1* (protein ID 184359) and *Lcr2* (protein ID 188627). Protein ID, gene name, motif identifiers, motif sequences, and motif and MAST scores are provided.
(XLS)

Table S8 **Primer sequences.** Excel file listing the sequences of the oligonucleotides used as primers for transcript profiling of TF and TR genes. Protein ID, transcript ID, gene family name, and forward and reverse primer sequences are provided.
(XLS)

Figure S1 **Expression level of marker genes *Cah1* and *Mca*.** (A) Cah1 and Mca expression levels were determined by measuring their transcript abundance by RT-qPCR. The median Ct values ± SD of the genes encoding actin, periplasmic carbonic anhydrase 1 (Cah1), and mitochondrial carbonic anhydrase (Mca) are shown demonstrating the relative stability of Actin expression during the shift from HC to LC. Actin was used as reference gene for determining relative Cah1 and Mca expression; the comparison of expression levels between the HC and LC conditions showed over-expression of Cah1 (log2, FC = 65) and Mca (log2, FC = 139) at low CO2 concentration. (B) Relative expression level of Cah1 after the shift from HC to LC shown as -ΔCt values. Error bars indicate standard deviation (SD) from the mean obtained in three biological replicates. (C) Relative expression level of Mca after the shift from HC to LC shown as -ΔCt values. Error bars indicate SD from the mean obtained in three biological replicates. Stars (*) indicate time points where significant changes relative to the 0 min time point were detected (p-value < 0.05; dependent t-test for paired samples).
(TIF)

Figure S2 **Reconstructed directed GRN of the CCM in *Chlamydomonas reinhardtii*.** Nodes of the network represent the genes and the edges containing arrows indicate the direction of the regulatory event. Numbers indicate protein IDs. File format: tif.
(TIF)

Figure S3 **Test of crosslinking efficiency and DNA recovery from crosslinked and de-crosslinked FAIRE samples.** The efficiency of the crosslinking procedure was assessed by separation of the chromatin samples on 1% agarose gels. (A) Samples of non-sonicated chromatin crosslinked for 5 min and de-crosslinked overnight at 65°C were subjected to standard DNA extraction using phenol-chloroform. As can be seen in lane 1, de-crosslinked DNA was recovered from the aqueous phase by the DNA extraction procedure. However, when de-crosslinking was omitted after the crosslinking step, no genomic DNA was recovered in the aqueous phase (lane 2), as expected. (B) and (C) Effect of crosslinking time on sonicated chromatin samples. In (B), non-crosslinked chromatin (lane 1) and chromatin crosslinked with 1% formaldehyde for 1 min (lane 2), 2 min (lane 3) and 5 min (lane 4), but not de-crosslinked, is shown (DNA taken from the aqueous phase after phenol-chloroform extraction). As shown in (C), de-crosslinking of sonicated FAIRE samples allowed efficient recovery of DNA by phenol-chloroform extraction from samples previously crosslinked. Most efficient recovery of DNA fragments was achieved when cells were crosslinked for 5 min before the de-crosslinking (lane 4). M, molecular weight marker. Labels (+) and (-) indicate whether crosslinking (´cross´) or de-crosslinking (´de-cross´) was applied or not.
(TIF)

File S1 **CCM_IOTA_GRN**.
This file contains the information about the nodes and node attributes of the reconstructed gene regulatory network of the CCM in *Chlamydomonas*, which can be explored in a dynamic manner. This file can be visualized with the open source bioinformatics software platform Cytoscape (http://www.cytoscape.org).
(TXT)

File S2 **Subnetwork CCM.** This file includes the SBML-based model for a subnetwork of genes discussed in the manuscript; we note that the model includes the simplest form of kinetics (i.e., mass action) without specifying values for the (assumed) kinetic parameters.
(XML)

File S3 **Edgelist**.
This file presents the genome-scale model of gene regulation in a text format containing the paired genes (regulator and regulated).
(TXT)
