# Correction: Genome-Wide Identification of Regulatory Elements and Reconstruction of Gene Regulatory Networks of the Green Alga *Chlamydomonas reinhardtii* under Carbon Deprivation

**DOI:** 10.1371/annotation/63d1c5f9-82c8-406b-b60e-b7d9ecba9408

**Published:** 2014-01-06

**Authors:** Flavia Vischi Winck, Samuel Arvidsson, Diego Mauricio Riaño-Pachón, Sabrina Hempel, Aneta Koseska, Zoran Nikoloski, David Alejandro Urbina Gomez, Jens Rupprecht, Bernd Mueller-Roeber

The name of the first author was incorrectly expressed in the Citation. The correct Citation is: Winck FV, Arvidsson S, Riaño-Pachón DM, Hempel S, Koseska A, et al. (2013) Genome-Wide Identification of Regulatory Elements and Reconstruction of Gene Regulatory Networks of the Green Alga Chlamydomonas reinhardtii under Carbon Deprivation. PLoS ONE 8(11): e79909. doi:10.1371/journal.pone.0079909. 

