# Supplementary figures and images for: Genome-Wide Identification of Regulatory Elements and Reconstruction of Gene Regulatory Networks of the Green Alga Chlamydomonas reinhardtii under Carbon Deprivation
Source: PLoS One. 2013 Nov 1;8(11):e79909. doi: 10.1371/journal.pone.0079909 (PMC3816576; doi:10.1371/journal.pone.0079909)

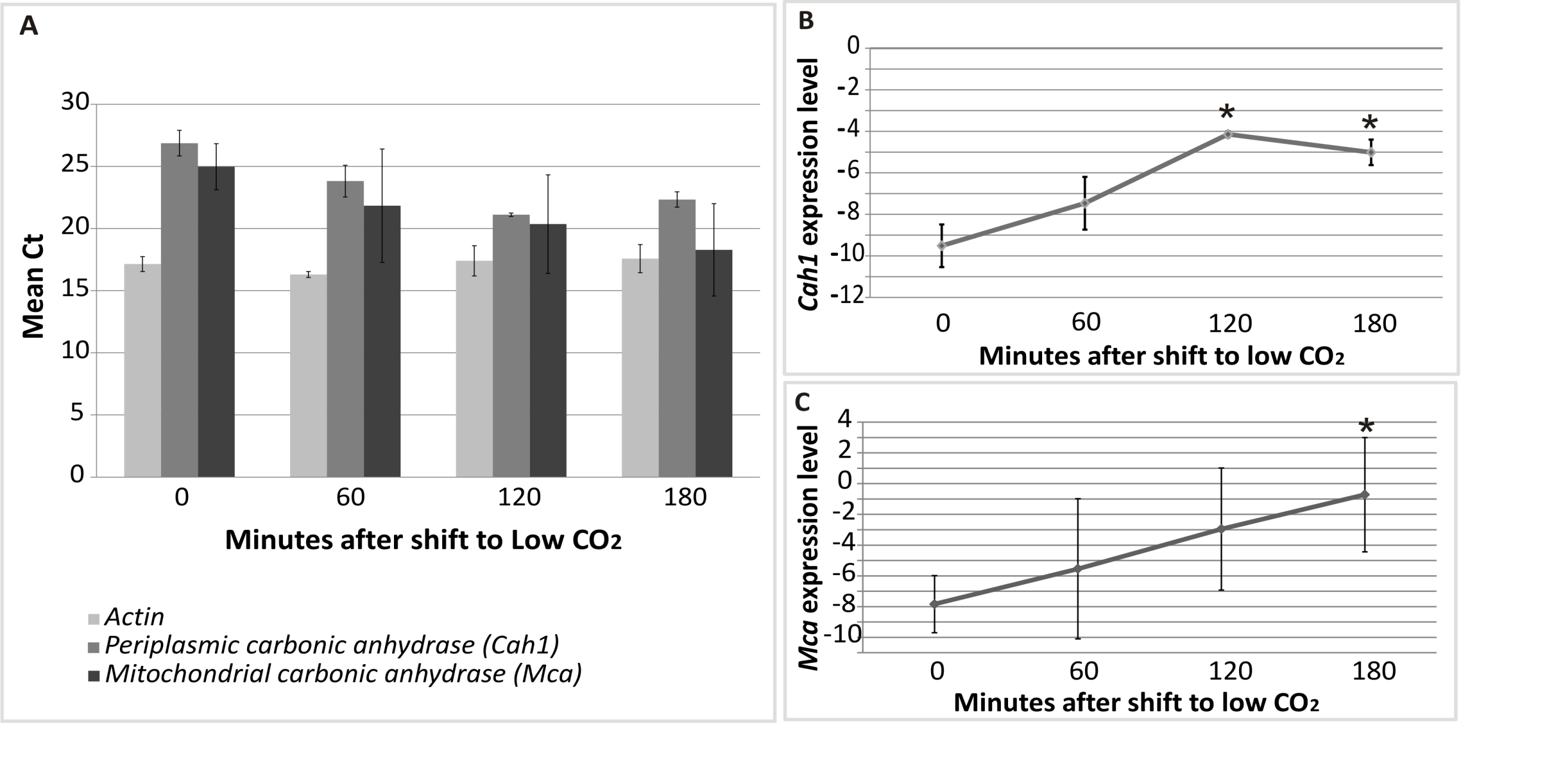

Supplement: Figure S1 — Expression level of marker genes Cah1 and Mca. (A) Cah1 and Mca expression levels were determined by measuring their transcript abundance by RT-qPCR. The median Ct values ± SD of the genes encoding actin, periplasmic carbonic anhydrase 1 (Cah1), and mitochondrial carbonic anhydrase (Mca) are shown demonstrating the relative stability of Actin expression during the shift from HC to LC. Actin was used as reference gene for determining relative Cah1 and Mca expression; the comparison of expression levels between the HC and LC conditions showed over-expression of Cah1 (log2, FC = 65) and Mca (log2, FC = 139) at low CO2 concentration. (B) Relative expression level of Cah1 after the shift from HC to LC shown as -ΔCt values. Error bars indicate standard deviation (SD) from the mean obtained in three biological replicates. (C) Relative expression level of Mca after the shift from HC to LC shown as -ΔCt values. Error bars indicate SD from the mean obtained in three biological replicates. Stars (*) indicate time points where significant changes relative to the 0 min time point were detected (p-value < 0.05; dependent t-test for paired samples). (TIF) [file pone.0079909.s009.tif]

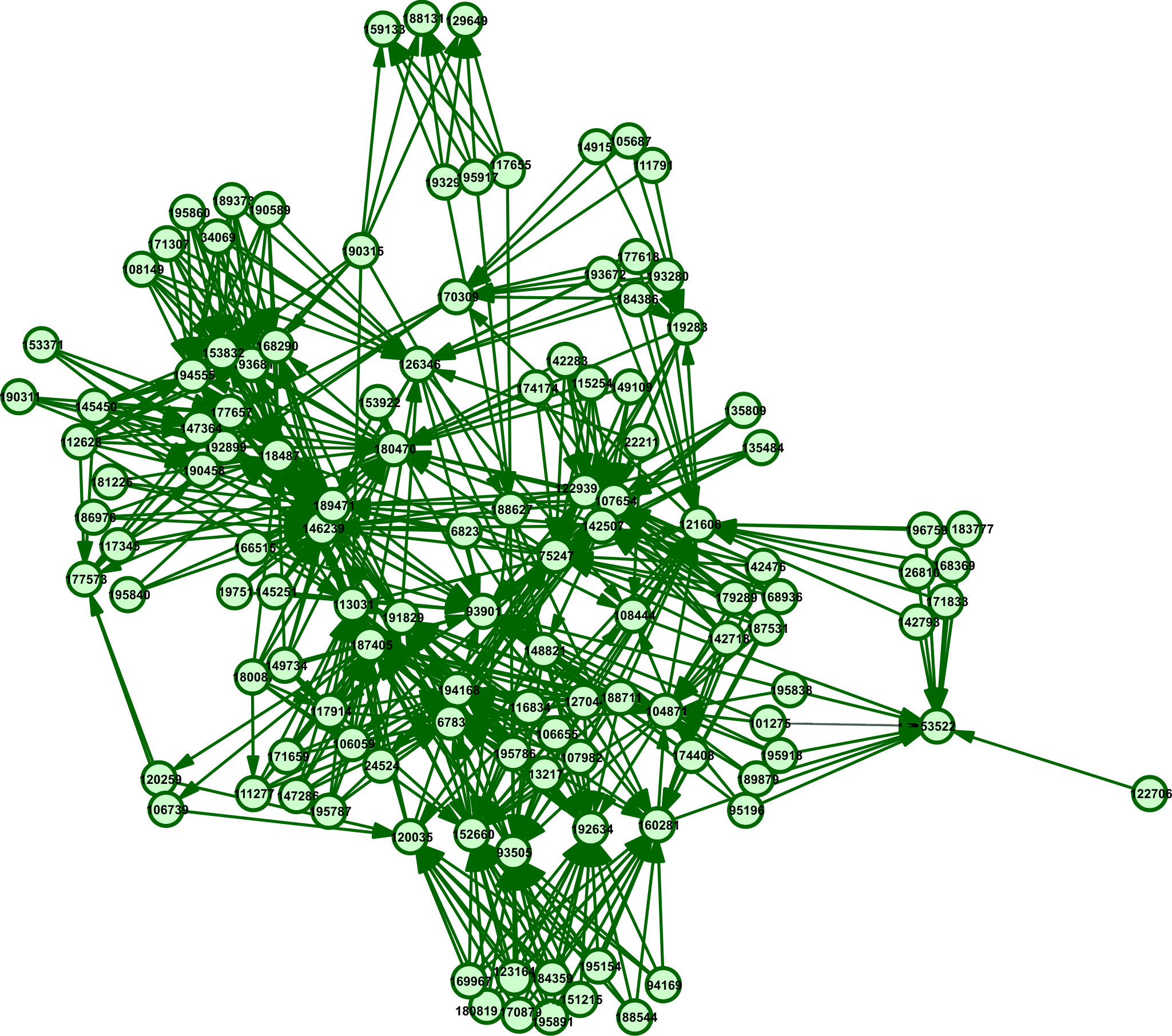

Supplement: Figure S2 — Reconstructed directed GRN of the CCM in Chlamydomonas reinhardtii. Nodes of the network represent the genes and the edges containing arrows indicate the direction of the regulatory event. Numbers indicate protein IDs. File format: tif. (TIF) [file pone.0079909.s010.tif]

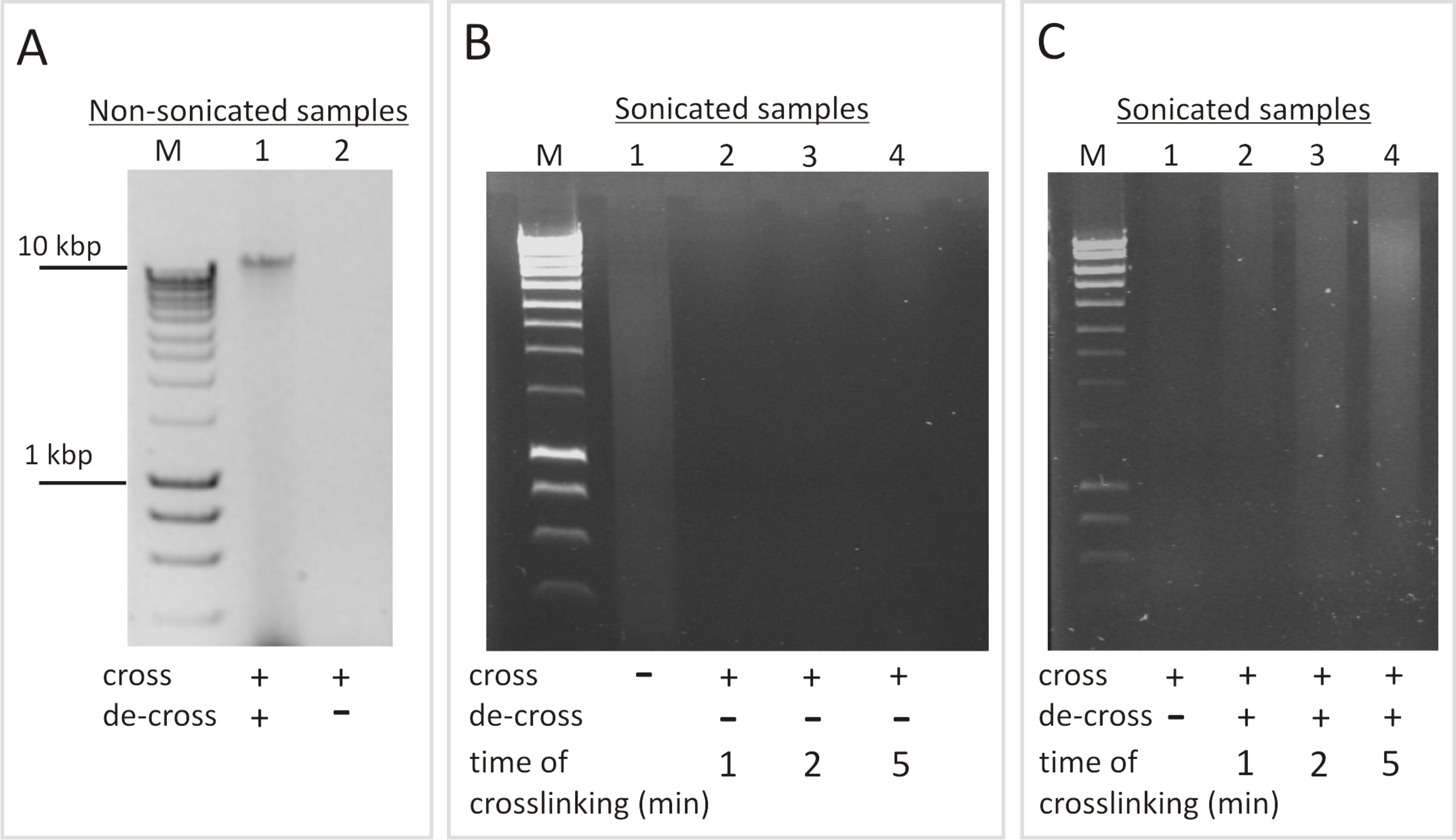

Supplement: Figure S3 — Test of crosslinking efficiency and DNA recovery from crosslinked and de-crosslinked FAIRE samples. The efficiency of the crosslinking procedure was assessed by separation of the chromatin samples on 1% agarose gels. (A) Samples of non-sonicated chromatin crosslinked for 5 min and de-crosslinked overnight at 65°C were subjected to standard DNA extraction using phenol-chloroform. As can be seen in lane 1, de-crosslinked DNA was recovered from the aqueous phase by the DNA extraction procedure. However, when de-crosslinking was omitted after the crosslinking step, no genomic DNA was recovered in the aqueous phase (lane 2), as expected. (B) and (C) Effect of crosslinking time on sonicated chromatin samples. In (B), non-crosslinked chromatin (lane 1) and chromatin crosslinked with 1% formaldehyde for 1 min (lane 2), 2 min (lane 3) and 5 min (lane 4), but not de-crosslinked, is shown (DNA taken from the aqueous phase after phenol-chloroform extraction). As shown in (C), de-crosslinking of sonicated FAIRE samples allowed efficient recovery of DNA by phenol-chloroform extraction from samples previously crosslinked. Most efficient recovery of DNA fragments was achieved when cells were crosslinked for 5 min before the de-crosslinking (lane 4). M, molecular weight marker. Labels (+) and (-) indicate whether crosslinking (´cross´) or de-crosslinking (´de-cross´) was applied or not. (TIF) [file pone.0079909.s011.tif]
